# The Lived Experience of Informal Caregivers of People Who Have Severe Mental Illness and Coexisting Long‐Term Conditions: A Qualitative Study

**DOI:** 10.1111/hex.14119

**Published:** 2024-06-16

**Authors:** C. Carswell, J. V. E. Brown, D. Shiers, R. Ajjan, A. Balogun‐Katung, S. Bellass, R. I. G. Holt, R. Jacobs, I. Kellar, C. Lewisohn, J. Lister, N. Siddiqi, I. Sidorova, P. Coventry

**Affiliations:** ^1^ Department of Health Sciences University of York York UK; ^2^ Psychosis Research Unit Greater Manchester Mental Health NHS Trust Manchester UK; ^3^ Division of Psychology and Mental Health University of Manchester Manchester UK; ^4^ School of Medicine Keele University Staffordshire UK; ^5^ Clinical and Population Sciences Department, Leeds Institute for Cardiovascular and Metabolic Medicine University of Leeds Leeds UK; ^6^ Population Health Sciences Institute, Faculty of Medical Sciences Newcastle University Newcastle UK; ^7^ Department of Sport and Exercise Sciences Manchester Metropolitan University Manchester UK; ^8^ Human Development and Health, Faculty of Medicine University of Southampton Southampton UK; ^9^ National Institute for Health Research Biomedical Research Centre University Hospital Southampton NHS Foundation Trust Southampton UK; ^10^ Centre for Health Economics University of York York UK; ^11^ Department of Psychology University of Sheffield Sheffield UK; ^12^ DIAMONDS Voice DIAMONDS Programme Patient and Public Involvement Panel York UK; ^13^ Bradford District Care NHS Foundation Trust Bradford UK; ^14^ Centre for Health and Population Sciences Hull York Medical School York UK; ^15^ York Environmental Sustainability Institute University of York York UK

**Keywords:** caregiver burden, informal caregivers, long‐term conditions, qualitative, severe mental illness

## Abstract

**Background:**

People with severe mental illness (SMI) experience higher rates and poorer outcomes of physical long‐term conditions (LTCs). The management of SMI and LTCs is highly complex and many people with SMI rely on informal carers for support, which may lead to high levels of caregiver burden, and caregiver burnout. Caregiver burnout can result in poor health outcomes for informal carers and a reduction in the quality of care they are able to provide. Therefore, it is important to understand the caring experience to identify and address factors that contribute to burden and burnout.

**Methods:**

This paper reports a secondary qualitative analysis of semistructured interviews and focus groups conducted with informal carers of people who have coexisting SMI and LTCs. We recruited 12 informal carers in England between December 2018 and April 2019. The transcripts were coded and analysed thematically.

**Results:**

We identified two overarching themes and five subthemes. The themes included ‘Fighting on all fronts: Mounting strain between demands and resources’, which described the challenge of providing care in the context of coexisting SMI and LTCs, and ‘Safekeeping: The necessity of chronic hypervigilance’, which captured how informal carers' roles were defined by managing high‐risk situations, leading to hypervigilance and paternalistic approaches to care.

**Conclusion:**

The experience of informal carers for people with SMI and coexisting LTCs is marked by limited access to support and the management of significant risk, which could contribute to high caregiver burden. Further primary research is needed to understand how the experiences of the caregiver role for people with SMI and LTCs influence caregiver burden.

**Patient or Public Contribution:**

Our PPI panel DIAMONDS Voice provided guidance on this study from conception, design and development of interview guides and recruitment materials to final write‐up. DIAMONDS Voice consists of service users and carers who have experience of SMI and LTCs. Three carer members reviewed the final manuscript, and two are credited as authors.

## 1 Introduction

People with severe mental illness (SMI; enduring psychiatric conditions that can present with psychosis, such as schizophrenia and bipolar disorder) [1] experience significant health disparities, on average dying 15–20 years earlier than people who do not have SMI [2]. This phenomenon is known as the mortality gap and is largely driven by higher rates and poorer outcomes of physical long‐term conditions (LTCs), such as diabetes and cardiovascular disease, among those with SMI [3].

There are many reasons for the inequity in health outcomes. The psychiatric medications commonly used to manage SMI, including antipsychotics and mood stabilisers, are associated with adverse effects such as increased appetite, fatigue, weight gain and metabolic syndrome [4, 5]. The symptoms of SMI, such as hallucinations and depression, can be highly distressing and may influence a person's ability to engage in healthier behaviours or lead them to engage in harmful behaviours, such as smoking, as a coping strategy [6, 7]. People with SMI are also more likely to experience social deprivation, isolation and poverty [8, 9, 10]. In addition, they may face significant barriers to accessing healthcare services, including stigma, diagnostic overshadowing and difficulty navigating fragmented healthcare systems [11, 12].

The management of SMI and LTCs is highly complex. Good clinical outcomes for LTCs are dependent on a person's ability to engage in self‐management [13]. Self‐management refers to day‐to‐day activities that are essential to maintaining health when living with an LTC, such as being physically active, adhering to medication and nutrition guidelines, monitoring physical symptoms and managing stress. However, people living with SMI can face significant barriers to self‐management, including the symptoms of SMI, the side effects of psychiatric medication, socioeconomic inequalities and stigma [7, 14]. As a result, many people with SMI require additional support managing their LTCs, including relying on unpaid, or ‘informal’, carers [6].

Informal carers are family members or friends who provide a wide range of typically unpaid support (although they may be in receipt of benefits as a result of their caring role) [15]. This role can involve managing medications, organising and supporting someone to attend appointments, attending to personal care, managing finances, encouraging engagement in health behaviours and monitoring their mental and physical health among other responsibilities [16]. Approximately 9% of residents in England and Wales provide unpaid or informal care to people with long‐term mental or physical health conditions [17], with 1.5 million caring for people with a mental health problem [18]. Informal caring is associated with a high level of caregiver burden. Caregiver burden can be defined as the overall multifaceted strain experienced by informal carers over time, which impairs their ability to provide care [19, 20]. This can be driven by a lack of resources, financial precarity, lack of support and multiple, competing responsibilities [19, 21]. Approximately 41% of informal carers of those with SMI experience severe burden [22], and caregiver burden is higher among carers supporting people with a psychiatric illness, compared to those supporting people with a long‐term physical illness [23].

Caregiver burden is associated with burnout [24]. Burnout is the impact of role strain on a person's well‐being and consists of three main dimensions: emotional exhaustion (the depletion of a person's psychological resources), depersonalisation (detachment from the beneficial impact of their caring role on others) and reduced personal accomplishments (a diminished or absent sense of achievement) [25]. Burnout can lead to several negative consequences [26], including impacting the carer's own health and well‐being. Approximately 29% of informal carers' experience depression [27, 28], which is significantly associated with caregiver burden and burnout [29]. Burnout can lead to a reduction in care provision, meaning carers reduce the amount of care they provide, or step away from their caregiving role entirely [19].

Caring for people with both SMI and LTCs presents unique challenges owing to high caregiver burden associated with informal care and the complex interplay between SMI and LTCs. While there is existing qualitative research that includes informal carers' perspectives in the management of LTCs among people with SMI, there is less research focusing on carers' own experiences of their role in supporting people with coexisting SMI and LTCs [30].

### 1.1 Aim

The aim of this study was to explore the experiences of informal carers of people who have SMI and coexisting LTCs.

## 2 Methods

The data reported here were collected as part of the DIAMONDS programme [31] qualitative exploration of the self‐management of LTCs among people with SMI and LTCs (‘DIAMONDS Quest’). The study involved interviews and focus groups with service users, informal carers and healthcare professionals, and the findings have been reported separately [6]. While the initial analysis focused on the experience of self‐management, a secondary analysis of the data was conducted, focused on the data collected from informal carers to explore the experience of providing care to people with SMI and coexisting LTCs, within the framework of caregiver burden.

### 2.1 Study Design

We undertook a qualitative study using focus group discussions and semistructured interviews with informal carers of people with SMI and coexisting LTCs.

### 2.2 Ethical Approval

The study was approved by the North West—Greater Manchester West Research Ethics Committee (REC reference: 18/NW/0603). Voluntary informed consent was obtained from all participants.

### 2.3 Setting and Recruitment

Participants included informal adult carers of people who had a diagnosis of schizophrenia, schizoaffective disorder, bipolar disorder or other nonorganic psychosis, alongside a coexisting cardiovascular, metabolic and/or respiratory LTC. Informal carers included spouses, partners, family members and friends who may or may not live with the person who they support.

Informal carers were recruited across England. Carers were identified through service users who had also participated in interviews as part of DIAMONDS Quest [6], and through carers' groups that were linked to primary care sites, mental health trusts and third sector organisations acting as recruiting sites in DIAMONDS Quest [6]. The study was also advertised on posters and flyers aimed at informal carers.

### 2.4 Data Collection

We recruited 12 informal carers and conducted five one‐to‐one semistructured phone interviews and two focus group discussions with informal carers (with four and three participants, respectively) between December 2018 and April 2019. The interviews and focus groups were conducted by one of three researchers, all of whom had experience in collecting qualitative data. An overview of the participants can be found in Table 1.

**Table 1:** Characteristics of interview and focus group participants.

Interview/focus group number	Sex	Relationship with the person they care for
Interview 1	Male	Father, caring for their daughter
Interview 2	Female	Mother, caring for their son
Interview 3	Female	Sister, caring for their sister
Interview 4	Female	Mother, caring for their son
Interview 5	Male	Caring for multiple people (caring for their brother, father and friend)
Focus group 1		
Participant 1	Female	Partner, caring for their male partner
Participant 2	Female	Partner, caring for their male partner
Participant 3	Male	Father, caring for their son
Participant 4	Female	Sister, caring for their sister
Focus group 2		
Participant 1	Male	Husband, caring for their wife
Participant 2	Male	Husband, caring for their wife
Participant 3	Female	Mother, caring for their son

The interviews and focus group topic guides were created to explore the carer's experiences of supporting a person with SMI and LTCs to manage their physical health. The topic guides were developed and refined in collaboration with our Patient and Public Involvement (PPI) group, DIAMONDS Voice. DIAMONDS Voice consists of service users and carers who have experience of SMI and LTCs, who meet three to four times a year to provide input on all aspects of the DIAMONDS programme.

### 2.5 Data Analysis

The focus group discussions and semistructured interviews were audio recorded with permission from all participants and transcribed verbatim. Transcripts were anonymised and imported into NVivo12 [32]. The transcripts were initially analysed to understand the experience of self‐management for people with SMI and coexisting LTCs [6]. However, after the initial analysis, it became apparent that the carer transcripts provided a rare and in‐depth insight into the caring experience and reflected the challenges of caring for people with SMI. Therefore, a thematic analysis [33] was carried out that focused exclusively on the experiences of informal carers. This analysis was underpinned by a critical realist epistemology, acknowledging the reality of the social world carers live within, and the mechanisms that contribute to individual experiences [34].

The transcripts were coded inductively to generate a set of codes that reflected the focus of this secondary analysis. All coding and initial analyses were conducted by C.C., a postdoctoral research fellow and mental health nurse, with experience in qualitative research, who was also involved in the original analysis for DIAMONDS Quest [6]. The codes were reviewed and arranged into higher order codes. The higher order codes were then organised into overarching themes, guided by the objective of the study and the informal caregiving integrative model. The informal caregiving integrative model is a theoretical framework that was developed to understand the consequences of the caregiving role. The framework captures different determinants, mediators and outcomes of the caregiving role [15]. The themes and subthemes were then reviewed by J.V.E.B., P.C., and D.S. before being finalised.

## 3 Results

Two overarching themes were identified during the analysis, including a total of five subthemes. Table 2 provides an overview of the themes and subthemes, with additional descriptors.

**Table 2:** Themes and subthemes.

Themes	Description of themes	Subthemes
Fighting on all fronts: Mounting strain between demands and resources	Focused on how the interaction between SMI and LTCs places high demands on carers' energy and time, with limited access to services and support	Navigating the SMI‐LTC maze
		Temporal tugs of war
		The complex battlefield of the caregiver role
Safekeeping: The necessity of chronic hypervigilance	Captures how carers appraise and manage the risk that underpins their caregiving role	Unremitting risk management
		Default to paternalism

### 3.1 Fighting on all Fronts: Mounting Strain Between Demands and Resources

Carers described how the interaction between SMI and LTCs has a multiplier effect on the challenges of the caregiving role. This reflects an increase in treatment burden, difficulties navigating fragmented services within the healthcare system and interaction between the symptoms of SMI and LTCs compounding the impact of each condition. Cognitive impairments and treatment side effects such as sedation also contribute to the challenges.

### 3.2 Navigating the SMI‐LTC Maze

A high number of medical assessments, appointments and multiple medications contribute to the increased treatment burden. There was a feeling among carers that if the person they cared for did not have an SMI, they would be better equipped to address the demands of their physical health condition independently.

I think things like they don't understand their illness, they don't understand their medication, they don't understand how the paperwork, financial stuff, works, the letters, they don't; even normal people can't deal with things like that so when you've got an illness you, it makes it even harder, doesn't it?

—Interview 5

Carers found navigating the relationship between SMI and LTCs significantly challenging. Carers described how LTCs and SMI adversely interact with each other with both symptoms and disease impact, recognising this interaction adds to the unpredictable nature of their conditions. For example, one participant highlighted how liver disease could exacerbate symptoms of SMI at any time, due to neuropsychiatric symptoms.

Anything could happen with his mind or can just go like, cos the liver's linked to everything so it can cause problems in the mind and the brain and everything, if that's got a problem then everything's got a problem, basically.

—Focus group 1, participant 1

This interaction can complicate the experience of seeking treatment within the healthcare service where carers perceive specialisms as compartmentalised and fragmented. This was described as a ‘Catch‐22’, as the LTCs exacerbate symptoms of SMI, yet access to services for LTCs may depend on resolution of SMI symptoms. Carers reported that healthcare professionals declined to provide treatment for a LTC until the symptoms of SMI resolve, and as a result feel trapped within a healthcare system that does not enable the people they care for to receive necessary, timely treatment,

Participant 1: About the physical and the mental being connected. And if the mental isn't treated or isn't dealt with then it can also stop them from doing operations that my wife requires. So, she requires an operation which is a major operation but because she's mentally ill, they won't do that operation ‘til her mental illness is sorted but the physical is also affecting the mental because she can't do what she wants to do.

Participant 2: Yeah. Catch 22.

—Focus group 2

#### 3.2.1 Temporal Tugs of War

Carers stressed that support was essential to provide them time to pursue their own relationships and interests separate from the caring role, which was crucial to coping with the associated demands. Typically support came from informal sources, such as family members. The need for support was related to the time‐intensive nature of caring. Spreading the responsibility across multiple people lessened the burden on the primary caregiver,

Well, I've got family members, friends, and we all work together, we have a rota, you know, what needs to be done every day so we kinda work together, cos one person can't do everything.

—Interview 5

The benefit of sharing the time commitment across multiple people was highlighted by carers who did not have a wide support system. Time was a precious commodity that was needed to maintain their own well‐being. They described struggling to find even small amounts of time for themselves, as their caring role was all‐consuming,

But I am trying to get some help like just come in to do, prepare a meal for her, what might give me just an hour off so I could just do what I wanted to do.

—Interview 3

Carers lacking a ready‐made support network often felt isolated, leading them to seek solace and assistance from formalised support centres,

The Carers Centre's been a great safe haven for me and it's kinda saved me (laughs) cos I didn't know where I was going before that. I was very isolated with him so we was just all by ourselves.

—Focus group 1, participant 1

Support systems were crucial to facilitate access to free time; however, time was also essential for nurturing social support networks. Carers described how providing around‐the‐clock support resulted in isolation,

I lost a lot of friends because I didn't feel I was able to go and be with friends. Because that time, I felt his need was more than mine. It's 24/7.

—Focus group 2, participant 3

The sense that time slips away for carers was also triggered by their encounters with the healthcare system. Carers recounted the challenges faced by those they cared for, highlighting extended waiting periods for crucial treatment referrals, with waiting lists for essential support lasting up to a year. Furthermore, they expressed frustration with waiting for timely access to healthcare professionals who would take their concerns seriously:

Everything takes so long! And by the time, you know, if there is a problem it should be nipped in the bud not left for months and months and years in fact.

—Interview 1

#### 3.2.2 The Complex Battlefield of the Caregiver Role

The experience of caring for someone with SMI and coexisting LTCs was consistently framed using the metaphorical language of fighting and war. These metaphors were predominantly used in the context of SMI, demonstrating the primacy of SMI, compared to LTCs, in the caring role. These allusions to being engaged in battles extended to multiple aspects of caring. For example, carers described how trying to support the person they cared for to engage in basic self‐care tasks, even eating and drinking, was framed as a battle against the symptoms of SMI,

I have known days where a culmination of his anxiety and hallucinations, it can be a battle just to get him to have a drink and a slice of toast.

—Interview 2

There were also legal battles that carers had to engage in, to ensure the person they cared for was receiving safe, high‐quality care. As living with SMI can result in a loss of legal rights, including detention under mental health legislation, this meant that numerous carers had the experience of acting as an advocate, at times in tribunals where legally binding decisions about their care were being made. This was also perceived as a battle that they had to undertake to keep the person they cared for safe,

When she was coming out of the mental hospital after you've been in there for five months they wanted to send her to a private hospital in [place] that's been on the television a few weeks ago, it's really bad, and it was only me going to the tribunal persuaded them not to send her there, cos if they'd have sent her there she wouldn't be here today, I'm convinced of that. So, you know, it's a battle, it's a battle all the time and it has been for a long, long time, twenty years anyway, because, as I say, when, it really got bad when she was in her teens, the violence.

—Interview 1

The use of analogies related to battles and fighting also stemmed from instances where individuals providing care sensed that their perspectives and ‘lived knowledge’ were disregarded. They felt that their role as a caregiver, along with their understanding of the complex physical and mental health needs of the person under their care, was often overlooked. This trend was observed consistently in both mental and physical health services, suggesting a broader cultural issue within healthcare that undervalues the contributions of informal caregivers.

Basically, a carer is only there just to change bedpans or to push them around or whatever, they haven't got any particular talent or … any knowledge and stuff, you know, and you're not treated as valued at all, your opinion doesn't matter, and yet you are often the closest person there …

—Focus group 1, participant 3

The constant struggle and experience of being undervalued led to feelings of helplessness that accumulated over time, leading to feelings of despair. Disempowered in the face of SMI, and perceived indifference from professional services, carers can lose any sense of hope,

So, I've got this ongoing problem for ten years now and I, frankly, I can't see any light at the end of the tunnel.

—Focus group 1, participant 3

### 3.3 Safekeeping: The Necessity of Chronic Hypervigilance

Informal carers described the high level of risk that the person they cared for faced, meaning a core component of their caring role was keeping them safe. This was predominantly driven again by the nature of SMI, which frequently necessitated prioritising the immediate safety needs over considerations for their long‐term health.

#### 3.3.1 Unremitting Risk Management

Carers described feeling a high level of risk and uncertainty that marked their day‐to‐day lives. One key risk was malnutrition and dehydration from self‐neglect. Carers described how people with SMI struggle to engage in the world around them, neglecting their health, their hygiene and activities of daily living. As a result of this risk, many carers felt the person they cared for could not live independently. This was further compounded by the perceived lack of support from services. For example, one carer recalled an experience shortly after their son was first diagnosed, when he was discharged from the hospital and living alone in another area,

My son, when he was first diagnosed, he wasn't living in this area at the time, and it was a friend of his who called me and said he wasn't very well. And I phoned and asked him what was wrong, and he wasn't specific. So, I went down to visit. I hadn't seen him in six months and the change that I found; I was appalled. Absolutely appalled at the state he was in and yet, he was then under the mental health team already which he hadn't told me about … . I was really appalled at the state he was in physically and mentally, obviously. And he was continually having fits.

—Focus group 2, participant 3

Another key source of risk that carers had to constantly evaluate and manage was self‐harm and suicide. Carers described how the people they cared for had repeated suicide attempts and episodes of self‐harm. Some of these episodes led to severe bodily injury,

She changed her medication, she poured petrol over her hand and set light to it, and I got a call from [place name] from her saying they're gonna take my hand off; that's how bad it was.

—Interview 1

As a result, carers had to constantly monitor the mood of the people they cared for, as their safety was constantly precarious. There was an awareness that seemingly small disruptions, disappointments or changes that the person they cared for experienced could result in self‐harm or further suicide attempts. Therefore, they constantly were trying to identify and mitigate against this risk wherever possible. However, once a risk was identified and heightened, carers experienced significant barriers in accessing essential services. At times this resulted from the profound stigma and diagnostic overshadowing, and the complexity of navigating both mental and physical health services. Not only did carers have to identify and mitigate risk but they also had to advocate and push for help to prevent serious harm,

Well, she has, she's, went downhill with diabetes, you know; I had three ambulances and two doctors and none of them diagnosed it … and she was sort of eight days without eating … so she almost died really.

—Interview 3

For some, this need for constant risk management and witnessing the person they cared for being significantly harmed was too difficult to cope with. To protect their own health and well‐being some carers decided that living with the person they cared for was no longer a sustainable option,

I mean she doesn't work, and we see her every day; she doesn't live with us because we couldn't handle the self‐harming. She nearly died a few years ago with taking a dose, overdose.

—Interview 1

#### 3.3.2 Default to Paternalism

Carers described how they had to default to a paternalistic caring style to manage risk and maintain the health of the person they cared for. This was heightened by the presence of LTCs, where carers had to strike a balance between mitigating short‐ and long‐term risks. Carers described how self‐management activities, such as exercise or eating healthy, which would mitigate the long‐term risks of LTCs, had to be pushed onto the person that they care for.

I make sure she has a healthy diet; so, I do all the meals every day. I try to get her just to go out, just for ten minutes, if I can, but she doesn't want to go but I have to keep pushing her to go, saying ‘Well you, it's good for you to get out’ and then she, eventually she will go, but she's soon back; and that's her daily routine at the minute.

—Interview 3

At times short‐term risks that resulted from the combination of LTCs and SMI also required a restrictive approach to support to reduce potential harm. For example, one carer described how access to sharp objects, including needles necessary to carry out blood glucose checks as part of routine diabetes self‐management, had previously been restricted to prevent episodes of self‐harm. This results in an increased burden on carers to manage behaviours typically associated with self‐management, such as monitoring blood sugar or administering medication,

Yeah, she does her blood checks every now and again, but then, it's only in the last six months that we've been able to do that because she was cutting herself with the needles …

—Interview 1

Other methods of control included limiting access to finances. Carers described restricting access to money for the person they cared for, to ensure that they were not able to purchase things that were harmful to their health.

I look after her money and I say ‘Right, you can take this much with you.’ To make sure she can't then go and get cigarettes and things like that.

—Focus group 1, participant 4

Other carer's described coercive techniques they would use to ensure that the person they cared for could meet their basic needs. These techniques were similar to ways parents may convince their children to do things that are good for them, but that they do not want to do. For example, one carer described how they would make sure the person they care for would eat,

I'm just gonna sit here, and I'm not gonna go home, until you eat something. Whether it's couple of slices of toast or whether it's a bowl of cereal, but I'm gonna sit here until you do. And because when he's in that zone, he doesn't like anybody around him, so he knows, to put it bluntly, the easiest way to get rid of me, and for him to go back on his own, is to actually—but he will literally come out of his room, he will eat the toast, he will have a cup of tea, and he will go back to his room.

—Interview 2

This aligns with how some carers viewed their role. Many carers were parents, and they viewed their relationship with their child as they progressed into adulthood, as an extension of the parenting role.

It's a funny thing when people say you're a carer, you're actually just a mum and dad basically and you do the things that mums and dads do; you make sure you nurture, you make sure somebody's fed and watered, washed, clothes are done, they look good.

—Interview 4

The constant risk management and default to a paternalistic style of caring was an unremitting experience. As carers were obligated to perform multiple tasks to reduce the risk of harm to both the physical and mental health of the person they cared for, they experienced a profound sense of exhaustion:

Participant 4: I'm more emotionally drained a lot of times; say if I've had a bad evening before with her and then she goes out in the morning and she's still grumpy …

Participant 2: It's draining …

—Focus group 2

The stress and strain of caring while also performing other roles was never more keenly felt than during moments of crisis, which could lead to crippling consequences for the carers' physical health:

I had a bit of a stressful time at work, and she was in a bad way, I managed to get the crisis team in to take her into hospital; that night I had a heart attack. So, it does put a lot of pressure on carers.

—Interview 1

## 4 Discussion

This qualitative study explores the lived experience of informal carers of people with coexisting SMI and LTCs. The secondary analysis of data from the DIAMONDS Quest study provides insights into how these experiences contribute to burden and burnout among informal carers [15, 28].

Our findings underscore how informal carers must navigate higher demands of their caring role that coincide with increasingly limited resources. While the management of SMI alone imposes a high caregiving burden, the complex interplay between symptoms of multiple conditions exacerbates the challenges of living with multimorbidity [35]. In the context of SMI where individuals may have limited capacity to manage multiple competing priorities for their own care, this additional burden can fall to informal carers. Failure of healthcare services to engage with informal carers can amplify their sense of isolation and impotence to redress increasing demands [36]. This situation is further compounded by well‐established barriers to accessing healthcare services, including fragmented services [37], diagnostic overshadowing [12] and lack of integrated multidisciplinary care [38]. Consequently, demands on informal carers of people with SMI appear to increase with the presence of LTCs, while their access to resources and support becomes increasingly restricted.

The heightened challenge of managing complex care with limited resources led informal carers to rely on war metaphors to describe their experiences [39]. Criticisms have been raised regarding the use of war metaphors in healthcare narratives, particularly in the context of ‘fighting’ diseases [40]. This is due to the value judgements inherent in this language. War metaphors frame disease as a discrete adversary, viewing treatment as a form of violence while the patient can become collateral damage [41]. However, the battle metaphors used by informal carers were not directed towards a specific disease. Instead, these metaphors were used to describe their struggle to provide care. This included difficulties accessing services, effectively acting as an advocate and convincing the person they cared for to attend to basic self‐care. Rather than fighting against a disease, they are fighting to provide care [42]. They are also fighting for recognition of their caregiving contribution, their need for information and greater involvement in the care process [36]. Furthermore, for some informal carers of people with SMI, using violent metaphors might be more congruent with their experiences of witnessing self‐directed violent behaviour when the person they care for is unwell [43, 44, 45]. Due to this exposure to violence, informal carers of people with SMI are significantly more likely to develop PTSD compared to carers of people without SMI [46].

Time was identified as a crucial resource for informal carers, supporting previous qualitative research that has demonstrated the inherent temporal aspect of the caregiving experience [47]. Caregiving is accompanied by a restriction on a person's use of time. This restriction means that informal carers' struggle to continue hobbies, engage in relationships and a social life separate from their caring role, or even maintain employment [48]. This results in limited ‘temporal agency’, the ability to determine not only how time is used but how time is experienced and defined [47, 49]. Instead, time is defined and spent within the caring role, restricting opportunities for cultivating a separate life and identity. Participating in meaningful social roles is an important coping strategy to prevent burnout in carers. These activities contribute to informal carer's well‐being by preserving an individual sense of identity [50].

However, similar to our original study (which included service users and healthcare professionals), the caring burden of LTCs was secondary to the impact of SMI. This difference was most marked by the level of risk associated with the symptoms of SMI. The high level of risk that carers experienced resulted in hypervigilance, with carers having to continually monitor for sudden deteriorations in mental or physical health, incidents of self‐harm or suicide attempts. Chronic stress and fear among informal carers are associated with the risk of these moments of crisis, including the person they cared for going through alcohol and substance misuse, receiving crisis mental health treatment and the risk of suicide or harm from others [51]. A study by Kalhovde and Kitzmüller [52] described various sources of risk that exacerbated fear and stress in family members of people with SMI. These included the influence of their family member on other people in their life and the fear of violent attacks. A consequence of this level of risk is constant hypervigilance, a characteristic of the caring role which is associated with exhaustion [52].

The need to manage high levels of risk led to a reliance on a paternalistic style of caring.

Paternalism in the care of people with SMI remains a contentious issue, often viewed as an essential component of psychiatric care that must be carefully balanced with protecting autonomy [53]. Paternalism is particularly relied upon when a person lacks capacity, as prioritising autonomy may be detrimental to the ethical principles of beneficence and nonmaleficence [54]. Therefore, informal carers may feel it is necessary to restrict a person's autonomy to prevent harm. This can act as an extension of formal psychiatric care, where decisions around finances, medication, healthcare and activities of daily living may be moved from the person living with SMI to a healthcare professional. However, this form of paternalism can also be interpreted as an extension of a parental role [55]. Previous research has demonstrated that parental carers of adult children view their parental obligation as integral to their caring role. Yet, this parental obligation is associated with significant grief over the loss of what they expected for both their own future and the future of their child [55, 56].

The descriptions of the impact of the caregiving role underscore the high burden and significant risk of burnout in this population. Emotional exhaustion, a core component of burnout, is characterised by feeling exhausted, depleted and drained of both emotional and physical resources [57]. A crucial risk factor for the development of emotional exhaustion is pessimism, coupled with a feeling of not being in control [15, 57]. In our study, informal carers described a pervasive feeling of helplessness and futility in the face of their caring responsibility. This helplessness stemmed from the lack of observable improvement in the mental health of the person they care for, a sense of being dismissed by services and an inability to find hope for the future. While personal accomplishment is often viewed as a key positive dimension of a caring role [58], the inability to witness improvement and find hope can significantly contribute to emotional exhaustion [15].

This article describes our secondary analysis of data collected for the DIAMONDS Quest study [6]. This analysis focused on informal carers' experiences that did not fit within the original aims of the DIAMONDS Quest study. Therefore, this analysis allowed us to focus on the caregiver experience to understand how these experiences may contribute to caregiver burden and burnout. However, as the data was initially collected to address a different aim, key aspects of the caregiving experience may have been missed. Additionally, as the original aim of the study was focused on barriers to management of LTCs, there was a lack of exploration of the positive aspects of the caring role. This study also had a small sample size, and we did not collect comprehensive demographic data from participants, which limits the representativeness of the findings and does not allow for any comparisons across different sociodemographic characteristics [28].

## 5 Conclusion

In conclusion, this secondary qualitative analysis highlighted how coexisting SMI and LTCs may increase the demands placed on informal carers, while simultaneously diminishing their ability to access necessary support. However, further research is needed to develop bespoke supportive interventions and create policy changes that address the unique challenges experienced by these informal carers.

## Author Contributions

**C. Carswell**: writing–review and editing, writing–original draft, project administration, formal analysis, conceptualisation. **J.V.E. Brown**: writing–original draft, writing–review and editing, project administration, formal analysis, conceptualisation. **D. Shiers**: conceptualisation, writing–original draft, writing–review and editing, formal analysis, methodology, funding acquisition. **R. Ajjan**: conceptualisation, methodology, writing–review and editing, funding acquisition. **A. Balogun‐Katung**: conceptualisation, methodology, investigation, writing–review and editing. **S. Bellass**: conceptualisation, investigation, project administration, methodology, writing–review and editing. **R.I.G. Holt**: writing–review and editing, conceptualisation, methodology, funding acquisition. **R. Jacobs**: funding acquisition, conceptualisation, methodology, writing–review and editing. **I. Kellar**: conceptualization, funding acquisition, methodology, writing–review and editing. **C. Lewisohn**: conceptualisation, methodology, writing–review and editing. **J. Lister**: writing–review and editing, conceptualisation, methodology. **N. Siddiqi**: conceptualisation, methodology, funding acquisition, investigation, project administration, writing–review and editing. **I. Sidorova**: writing–review and editing, conceptualisation, methodology. **P. Coventry**: conceptualisation, methodology, funding acquisition, writing–review and editing, writing–original draft.

## Conflicts of Interest

D. Shiers is an expert advisor to the NICE Centre for Guidelines; the views expressed are the authors' and not those of NICE. R.I.G. Holt has received honoraria for speaker engagement, conference attendance or advisory boards from: Abbott, AstraZeneca, Boehringer‐Ingelheim, European Association for the Study of Diabetes, Eli Lilly, Encore, Janssen, Menarini, NAPP, Novo Nordisk and Omniamed, Roche and Sanofi. P. Coventry is partly funded by the NIHR Applied Research Collaboration Yorkshire and Humber.

## Data Availability

The data sets used and/or analysed during the current study are available from the corresponding author upon reasonable request. The data are not publicly available due to privacy or ethical restrictions.
